# Personality, Parasites, Political Attitudes, and Cooperation: A Model of How Infection Prevalence Influences Openness and Social Group Formation

**DOI:** 10.1111/tops.12175

**Published:** 2015-11-27

**Authors:** Gordon D. A. Brown, Corey L. Fincher, Lukasz Walasek

**Affiliations:** ^1^ Department of PsychologyUniversity of Warwick

**Keywords:** Personality, Parasite stress, Ideology, Agent‐based model

## Abstract

What is the origin of individual differences in ideology and personality? According to the parasite stress hypothesis, the structure of a society and the values of individuals within it are both influenced by the prevalence of infectious disease within the society's geographical region. High levels of infection threat are associated with more ethnocentric and collectivist social structures and greater adherence to social norms, as well as with socially conservative political ideology and less open but more conscientious personalities. Here we use an agent‐based model to explore a specific *opportunities‐parasites trade‐off (OPTO)* hypothesis, according to which utility‐maximizing agents place themselves at an optimal point on a trade‐off between (a) the gains that may be achieved through accessing the resources of geographically or socially distant out‐group members through openness to out‐group interaction, and (b) the losses arising due to consequently increased risks of exotic infection to which immunity has not been developed. We examine the evolution of cooperation and the formation of social groups within social networks, and we show that the groups that spontaneously form exhibit greater local rather than global cooperative networks when levels of infection are high. It is suggested that the OPTO model offers a first step toward understanding the specific mechanisms through which environmental conditions may influence cognition, ideology, personality, and social organization.

## 1 Introduction

What gives rise to different cultures, attitudes, and politico‐economic systems in different regions of the globe? What might cause such attitudes and systems to change over time, and what might speed up or slow down such change? Here we adopt the framework of the *parasite stress hypothesis*, according to which many core human values and cultural patterns at least partly reflect adaptive responses, by a behavioral immune system, to the threat of infectious disease (e.g., Schaller & Park, 2011; Thornhill & Fincher, 2014).

Although some of its applications remain controversial (e.g., Hruschka et al., 2014; Kuppens & Pollet, 2014; Pollet, Tybur, Frankenhuis, & Rickard, 2014), the parasite stress hypothesis offers one potential explanation for the differences within both individuals and societies that are often simply taken as a starting point by disciplines such as economics and psychology. Moreover, the hypothesis suggests mechanisms by which climate change may influence global cognition and social transitions. For example, Hodges et al. (2014) suggested that global climate change might delay by many years China's progress in reducing transmissible diseases, and according to the parasite stress hypothesis such delay might in turn influence the time course of changes in the country's social structures and organization.

However, there has been almost no computational specification of the cognitive psychological mechanisms that might underpin the relation between parasite stress and values or of how such cognitive mechanisms might lead to the emergence of different (e.g., collectivist rather than individualist) social structures. Here, we make some first steps toward such a specification.

We focus on the effects of parasite stress on political ideology, on personality, and on the consequences of individual differences in openness to out‐group interactions for the structure of mutually cooperative social groups. Specifically, we seek to understand the mechanisms that may underlie earlier findings that high levels of parasitic infection within a region are associated with less open personalities (e.g., Schaller & Murray, 2008; Thornhill, Fincher, Murray, & Schaller, 2010), greater xenophobia and in‐group orientation (see Fincher & Thornhill, 2012, for a review), and more conservative political voting patterns (e.g., Brown et al., 2015; see Fincher & Thornhill, 2012, for a review). Consistent with the more general parasite stress hypothesis, Brown et al. interpreted their results in terms of a simple quantitative *opportunity‐parasites trade‐off hypothesis* (OPTO). OPTO aims to quantify some of the benefits and costs to an individual of interacting (e.g., trading or mating) with individuals from different regions (members of out‐groups). The model suggests that the optimal point on this trade‐off depends on prevailing levels of infectious disease. The OPTO model can be seen as a simple mathematical instantiation of one of the core features of the more general parasite stress hypothesis.

In this article we illustrate, using an agent‐based model, the operation of the OPTO mechanism and show how the attitudes and values of rational individuals might come to differ as a function of infection‐related threat (parasite stress) within their environment. We also use the model to show how evolved social groups may come to be more inward‐looking and xenophobic when prevailing levels of infection are high.

### 1.1 The parasite stress hypothesis

According to the parasite stress hypothesis (e.g., Fincher & Thornhill, 2012; Schaller & Duncan, 2007; Thornhill & Fincher, 2014), levels of parasitic infection within a region may drive variability at both individual and societal levels. The avoidance of death or disability through avoidance of infection is a major evolutionary driving force. For example, until times that are relatively recent in evolutionary terms, almost 50% of children failed to survive to reproductive age (Volk & Atkinson, 2013), with the majority of deaths being due to infectious diseases.^1^

Local parasite types and hosts defense systems will typically be involved in a co‐evolutionary arms race, and so at any given time a host's immunological defenses will be specialized to be most effective against local parasite species (Thompson, 2005). To the extent that a host defense system is specialized locally, contact with out‐groups will be associated with an increased risk of exposure to infectious diseases against which there is no a priori immunity (Fincher & Thornhill, 2008). The relevant infection‐avoiding behaviors such as avoidance of strangers and high conscientiousness in, for example, food preparation, are assumed to arise from the operation of a “behavioral immune system” (e.g., Schaller, 2011). The behavioral immune system comprises adaptive psychological response mechanisms that are sensitive to cues that might predict presence of infectious pathogens and which respond with appropriate cognitive processing and affective reactions. Such a system is likely to be oversensitive—the cost of a false positive (avoiding non‐existent infection) is small relative to the possible cost (catching avoidable infection; Kurzban & Leary, 2001). In social terms, an important component of the parasite stress hypothesis is that high levels of infection may lead to ethnocentrism, xenophobia, distrust of different others, and conformity (e.g., Murray, Trudeau, & Schaller, 2011; Neuberg, Kenrick, & Schaller, 2011; Thornhill, Fincher, & Aran, 2009)—because such behaviors will reduce the likelihood of exposure to unfamiliar infections to which immunity has not been developed.

A range of evidence is consistent with the parasite stress hypothesis (see Thornhill & Fincher, 2014, for a review); key relevant data are reviewed below. Many of the associations between pathogen stress and individual or societal characteristics are, however, correlational in nature and are sometimes criticized for this. Longitudinal studies are difficult, reflecting in part the difficulty of obtaining accurate historical data on pathogen prevalence (although see Murray & Schaller, 2010). However, the relevant associations survive the introduction of various statistical controls. Direct causal evidence for the operation of a behavioral immune system, albeit over shorter timescales, can be found experimentally. For example, priming people with slides containing disease‐salient cues leads to increased feelings of between‐person avoidance (Mortensen, Becker, Ackerman, Neuberg, & Kenrick, 2010) and a classical immune system response (Schaller, Miller, Gervais, Yager, & Chen, 2010).

### 1.2 Parasites, personality, and political ideology

We follow Thornhill and Fincher (2007) and others in hypothesizing that, in evolutionary terms, social conservatism is associated with in‐group specialization while social liberalism reflects out‐group specialization.

A small number of studies have examined the relationship between political ideology and parasite stress directly, while rather more have examined the relationship of parasite stress to collectivism. Collectivism, in contrast to individualism, is associated with an emphasis on strong distinctions between in‐group and out‐group members, developing and maintaining in‐group relationships, suspicion and avoidance of out‐group members, and conformity to local customs and social norms (e.g., food preparation and hygiene practices). These behavioral tendencies can all be interpreted as mechanisms to reduce the probability of exposure to infectious diseases. The collectivism–individualism dimension is very similar to a conservatism–liberalism ideological dimension (Thornhill & Fincher, 2014), particularly when social rather than economic conservatism is concerned (Everett, 2013)—social conservatism is associated with, for example, negative attitudes to out‐groups and adherence to social norms.

Pathogen prevalence is positively associated with various measures of collectivism at the level of both countries (Fincher, Thornhill, Murray, & Schaller, 2008) and U.S. states (Fincher & Thornhill, 2012; although see Hackman & Hruschka, 2013; Hruschka et al., 2014). At the level of the individual, endorsement of collectivist values is associated with measures of sensitivity to disgust and concern with infectious disease (Clay, Terrizzi, & Shook, 2012). Similar positive associations are found between measures of political conservatism and disgust sensitivity (Inbar, Pizarro, Iyer, & Haidt, 2012; Terrizzi, Shook, & McDaniel, 2013; Terrizzi, Shook, & Ventis, 2010), with a range of related findings being consistent with the suggestion that social and religious conservatism may in part reflect strategies for avoidance of infectious disease (Terrizzi, Clay, & Shook, 2014; Terrizzi et al., 2013). Thus, experimentally inducing disgust can increase prejudice toward out‐groups (Terrizzi et al., 2010), and showing people primes related to cleanliness can influence political attitudes (Helzer & Pizarro, 2011).^2^ Experimental primes that induce disgust may, however, also increase concerns about fairness, leading to more left‐wing scores on measures of political attitudes that focus on economic inequality (Petrescu & Parkinson, 2014). Overall, these findings are consistent with the suggestion that some dimensions of political ideology are associated with pathogen‐related variables (such as disgust).

High regional levels of parasite stress are associated with authoritarian societies and authoritarian attitudes at the level of individuals (Cashdan & Steele, 2013; Murray, Schaller, & Suedfeld, 2013; Thornhill & Fincher, 2014; Thornhill et al., 2009, 2010; although see Pollet, 2014). Brown et al. (2015) examined political ideology at the level of U.S. states directly, using a measure based on voting patterns and interest group ratings of political candidates (Berry, Ringquist, Fording, & Hanson, 1998). It was found that state‐level political ideology in the 1960s and 1970s was correlated with levels of parasitic infection, with greater conservatism in states with higher parasite stress, even after the introduction of controls for state‐level inequality, per capita GDP, and levels of health spending per capita. However, the correlation reduced over time and by the 1990s had virtually disappeared. The reduction in effect over time was interpreted as evidence that the importance of infection in determining political ideology reduces with improvements in general wealth and health, perhaps because of reduced infection‐related child mortality with consequent relative increases in the adaptive importance of other behaviors. At least in the developed West, it is at least possible that the (very recent, in evolutionary time) reduction in infection‐related mortality is reducing the importance of parasite stress as an evolutionary force (cf. Alexander, 1990). The ideological position of U.S. elected politicians, as identified by their voting patterns (McCarty, Poole, & Rosenthal, 2006), was also found to be more conservative in high‐infection states. Brown et al. (2015) interpreted the results on political ideology, along with other results on personality, in terms of the OPTO model outlined below.

### 1.3 The opportunity‐parasites trade‐off hypothesis

The aim of the OPTO model that we develop here is to provide a quantitative underpinning to the idea that the possession of personality and ideological characteristics can be understood in part as reflecting optimal trade‐off between the costs and benefits of interacting with geographically (and hence, by assumption, immunologically) distant individuals and members of out‐groups (Thornhill & Fincher, 2014). Benefits include the possibility that inhabitants of more distant regions may have access to complementary natural resources (leading to the possibility of economic gains through trading), along with access to a more diverse mating pool and exposure to socially or economically efficient cultural practices. Such benefits should, in themselves, lead to a relatively open, liberal, trusting, and cooperative attitude toward out‐group members from different geographical regions.

According to the parasite stress hypothesis, however, there are also costs associated with out‐group contact—primarily in terms of the risk of exposure to new and dangerous infectious agents because individuals from out‐groups are likely to have different immunity and potentially carry unfamiliar infectious diseases (e.g., Fincher & Thornhill, 2012; Schaller & Duncan, 2007). According also to the specific OPTO hypothesis that we implement here, core components of both political attitudes and personality reflect choices of different points on the relevant trade‐off curve, such that an ideological position along a socially conservative to liberal dimension may partly reflect an ancestrally adaptive response to high or low levels of parasitic infection within an environment (parasite stress). Thus, a more socially conservative cognitive and social style may in part represent a response to a specific type of environmental threat, while left‐leaning/liberal ideology may in contrast partly reflect an openness to new ideas and interaction with out‐groups when infection‐related threat is reduced.^3^

Consistent with such a view, the liberal end of the political spectrum, involving acceptance of out‐groups and dissimilar others possibly at the expense of maintaining in‐group social relationships, is associated with novelty seeking and high scores on the personality dimension of openness (e.g., Carney, Jost, Gosling, & Potter, 2008; see Hibbing, Smith, & Alford, 2013; and Mondak, 2010, for reviews). Moreover, in terms of the “Big 5” dimensions of personality (McCrae & Costa, 1987), high levels of pathogen prevalence are associated with lower average openness and extraversion within regions (Schaller & Murray, 2008; Thornhill et al., 2010). It should be noted that many of the observed associations are at the level of regions and aggregates of individuals; there is of course no guarantee that analogous relationships will be seen when individuals are the unit of analysis (cf. the “ecological fallacy”; see, e.g., Kuppens & Pollet, 2014; Pollet et al., 2014). In the case of personality, for example, the high correlations between parasite stress and personality at the levels of countries and U.S. states are not mirrored by equally high correlations between individual differences in infection susceptibility and personality traits (Duncan, Schaller, & Park, 2009; Tybur, Bryan, Lieberman, Hooper, & Merriman, 2011; Tybur & de Vries, 2013). Moreover, as we note below, alternative explanations have been offered for specific findings. It nonetheless appears that, taking geographical units as the unit of analysis, the parasite stress approach offers one useful perspective on the origin of differences in openness as reflected in both personality and political ideology (see also Nettle, 2006).

Here, we show—using an agent‐based model—that rational, utility maximizing individuals will (given some simple assumptions about the mechanisms that underpin group formation) learn to interact preferentially with geographically local rather than geographically distant others when infection prevalence is high, and to interact preferentially with more distant others when infection prevalence is low. Geographical distance is in the network treated as a proxy for immunological distance (thereby simplifying reality, in which immunologically different individuals may co‐exist in a given region). Moreover, we show (following Gray et al., 2014) that groups of mutually cooperative individuals can form even in the absence of initial individual differences, and we demonstrate that the groups that form are more assortative (preferentially associating with nearby others) and xenophobic when levels of parasite stress are high.

A particular advantage of using an agent‐based model in the present context is that the dynamics of possible causal mechanisms can be examined in a simulated environment, and that the development of different types of mutually cooperative group structure can be explored under different simulated conditions without the need to travel backward in time. Agent‐based models (ABMs) of social dynamics have been widely used to examine how cooperative behavior and group formation develop over time as an emergent property within networks of locally interconnected agents (e.g., Newman, 2010; Schelling, 1971). ABMs may facilitate integration of cognitive, social, and evolutionary approaches (see Goldstone & Janssen, 2005, for a recent review from a cognitive science perspective). A long‐standing focus has been on the conditions under which cooperation occurs (e.g., Nowak, 2006; Nowak & Highfield, 2011).

## 2 An agent‐based model

A useful starting point is provided by Gray et al. (2014), who describe a model in which group structure may emerge spontaneously out of simple mechanisms of reciprocity and transitivity. We extend their model to include the possibility of agents receiving negative payoffs if they become infected due to cooperation with an agent who is infected with a disease to which they have not developed immunity.

Gray et al. (2014) examined the evolution of cooperation in a social network. Cooperation is modeled using a simple Prisoners' Dilemma game in which agents both receive a moderate positive payoff (here +1) if they both cooperate, but both receive a moderate negative payoff (here −1) if they both refuse to cooperate, that is, if they both defect. If, however, one player chooses to cooperate but the other defects, the cooperator receives a large negative payoff (here −3), while the defector receives a large positive payoff (here +3). We follow Gray et al. (2014) in assuming a network of agents, each of which has an “affinity” (varying between 0 and 1) with each other agent.^4^ The connections are symmetrical—if agent *i* has an affinity of 0.6 with agent *j*, the reverse is also the case (i.e., agent *j* has an affinity of 0.6 with agent *i*). The affinity parameter governs the probability that a randomly chosen pair of agents will decide to play together. It also separately governs the probability that each agent independently will choose to cooperate given that they have decided to play together.

Gray et al. (2014)'s model includes reciprocity (if two players cooperate, their affinity increases; if two players defect, their affinity reduces) and transitivity (if agent *i* changes its affinity to agent *j*,* i* may also change its affinity to other agents to become more similar to agent *j*'s own affinity to other agents). In intuitive terms, if my probability of cooperating with you increases, my probability of cooperating with people that you are already close to may also increase. Gray et al. (2014) showed that these simple mechanisms lead to the formation of sub‐groups of mutually cooperative agents within the network, even though there are no initial differences between the agents (all affinity values are initially set to 0.5).

The first simulations we report here do not make use of reciprocity or transitivity, although we return to them below. We first add to the Gray et al. (2014) model the assumption that agents are arranged in a spatial grid, and we assume that the network is fully connected such that every agent has the potential to cooperate with every other agent.^5^ The grid can be seen as geographical, in that agents who occupy distant locations on the grid represent individuals who live in different regions. The distance between any two agents is simply the city‐block distance between their locations on the grid, and to avoid edge effects we assume that the grid “wraps round” such that the edges join onto each other. We then construct the environment to allow us to implement and explore the OPTO model. Two key assumptions are implemented; we term these the *opportunities assumption* and the *infection assumption*.

### 2.1 The opportunities assumption

First, it is assumed that the payoff that can be gained by each of two agents if they both cooperate increases as a function of the spatial distance separating them. This is intended to capture the intuition that agents distant from one another are more likely to have complementary natural, biological, or social resources, which can lead to increased economic or other fitness as a result of trading or mating. This distance‐related advantage of cooperation is specified as follows:

(1) $$R_{C_{i}C_{j}}=1+P*\frac{\left(d_{i,\;j}-1\right)}{\left(\max\_d-1\right)}$$

where $R_{C_{i}C_{j}}$ is the reward obtained by each of *i* and *j* if they cooperate, *d*_*i,j*_ is the distance separating them, and max_*d* is the longest distance between any pair of agents in the network. *P* is a parameter that specifies one aspect of the pay‐off matrix; here it is set to 9. The effect of this is that agents who interact (i.e., play the Prisoners' Dilemma game) with other (“out‐group”) agents from more distant locations have a chance of receiving a higher pay‐off if both agents cooperate. (If one or both agents defects, the payoffs they receive are unaffected by the distance separating them.)

### 2.2 The infection assumption

A second feature of our simulated environment is designed to capture the idea that the probability of one agent becoming infected with a disease to which they do not have immunity as a result of interacting with another agent increases with the distance between them (i.e., because they are assumed to be less likely to have developed immunity to such a disease). We refer to such infection as exotic infection, to indicate that we are concerned only with the probability of becoming infected with a disease to which immunity has not been developed. Thus, we do not model “regular” infection of the type likely to occur even between in‐group members occupying geographically close locations, nor do we model here the spread of infection throughout a social network.

Specifically:

(2) $$p\left(\inf_{i}\right)=p\left(\inf_{j}\right)=k*\frac{\left(d_{i,\;j}-1\right)}{\left(\max\_d-1\right)}$$

where *p*(inf_*i*_) is the probability of agent *i* becoming (exotically) infected, *k* is a parameter that specifies the probability of exotic infection at the maximum distance, and *d*_*i,j*_ and max_*d* are as before. If an agent becomes (exotically) infected, the agent receives a large single negative payoff (−50). The effect of this is that interacting with distant agents is risky because of the associated probability of exotic infection. Interaction with an immediately adjacent network neighbor always carries zero risk of exotic infection.^6^

The second key parameter that we investigate by simulation (i.e., in addition to varying *k*) is a characteristic not of the environment but of individual agents, and we refer to it as an agent's *openness*. The openness of an agent, which is constrained to be between −1 and +1, describes the relative willingness of the agent to interact with faraway rather than nearby other agents. The openness of an agent *i*,* O*_*i*_, determines its probability of interacting with another agent as a function of the geographical distance separating them as follows:

(3) $$p\left({}_{i}\text{play}{}_{j}\right)=\frac{\left(1-O_{i}\right)}{2}+O_{i}*\frac{\left(d_{i,\;j}-1\right)}{\left(\max\_d-1\right)}$$

where *p*(_*i*_play_*j*_) is the probability that agent *i* will play the Prisoner's Dilemma game with agent *j*, and the other parameters are as specified above. The effect is that agents with a high openness parameter will be more likely to interact with a faraway agent and less likely to interact with a nearby agent, while the reverse will be the case for an agent with low openness.

To illustrate: suppose an agent *i* has an openness (*O*_*i*_) of 1, and that the maximum spatial distance that can separate any pair of agents in the network is 11. The agent is given the opportunity to play with a maximally distant other agent (*d*_*i,j*_ = 11). The probability of *i* deciding to play with *j* will then (by Eq. (3)) be 1.0 (i.e., (1−1)/2 + 1*(11−1)/(11−1)). If in contrast agent *i* is given the opportunity to play with an immediate neighbor (*d*_*i,j*_ **= 1), the probability of *i* deciding to play with *j* will be zero (i.e., (1−1)/2 + 1*(1−1)/(11−1)). If instead agent *i* has “neutral” openness (*O*_*i*_ = 0), then the corresponding probabilities of play will be 0.5 (i.e., (1−0)/2 + 0*(11−1)/(11−1)) if the other agent *j* is maximally distant, and will again be 0.5 (i.e., (1−0)/2 + 0*(1−1)/(11−1)) for an immediate neighbor. Finally, if agent *i* has “negative” openness (*O*_*i*_ = −1), the play probabilities will be zero for a maximally distant other agent (i.e., (1 + 1)/2 −1*(11−1)/(11−1)), and 1.0 for an immediate neighbor (i.e., (1 + 1)/2 − 1*(1−1)/(11−1)). Thus, a highly open agent will increase its probability of interacting with another agent from 0 to 1 as the distance between them increases, while the reverse will occur for an agent with low openness.

Each agent independently decides whether to interact according to Eq. (3); they play only if both choose to do so. Note that the openness parameter simply governs how likely agents are to interact; it has no effect on the probability that they will cooperate if they do interact (that probability being given by their affinity). We view the parameter as capturing an important component of openness and social liberalism.

*Simulation 1: Openness and parasite stress*. We first illustrate the basic principles of the OPTO model by showing that the average payoffs received over multiple interactions for agents with different openness levels (O_*i*_ values) depend on the infection probability prevailing within the environment (given by the *k* parameter in Eq. (2)). We simulate a 20 × 20 grid of agents. Each agent is initialized with a random openness parameter (uniform in the interval [−1, 1]), and in the present simulation their openness does not change through a simulation. Each agent is given an affinity of 0.5 to every other agent; this also remains constant in this first simulation. Separate simulations are conducted for different values of the infection probability parameter *k*. The expectation is that high openness will be the best strategy (i.e., the strategy that will lead to higher payoffs in the long run) when the environmental probability of infection is low, while low openness will be the best strategy when environmental probability of infection is high.

Each round of a simulation works as follows. First, a distance is chosen at random from all the possible distances in the network.^7^ Second, two agents that are separated by that distance are chosen. The agents are chosen at random, but with an adjustment to reflect the fact that there are different numbers of agent‐pairs associated with different distances and to ensure that each agent has an equal opportunity of being selected in the long run. Third, each of the two agents independently decides whether to interact with the other, as determined by its individual openness and Eq. (3). If one or both players decides not to interact, the round ends there. If both players decide to interact, they play a Prisoners' Dilemma game, each cooperating with a probability determined by their mutual affinity (here constant at 0.5) and receive the appropriate payoff. For each of five different values of the infection probability, 100,000 rounds, each incorporating the steps just enumerated, were run.

**Figure 1:**
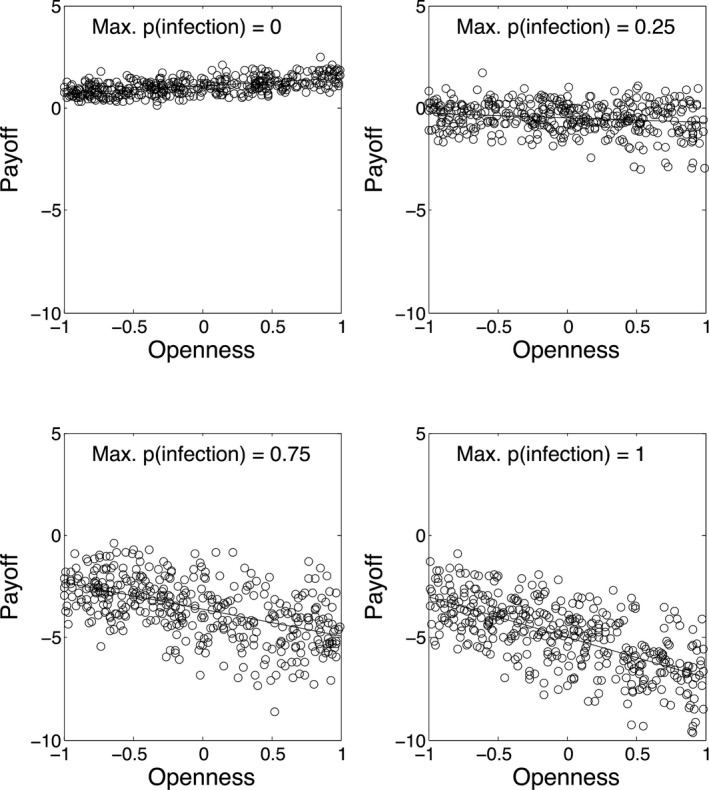
Effects of openness on payoffs/fitness as a function of probability of infection when interacting with the most distant neighbors.

The results are shown in Fig. 1, where it can be seen that the behavior of the model is as expected. The top left‐hand panel indicates the total payoffs received by agents of different openness when there is no probability of infection due to interacting with other agents. It is evident that there is a small fitness advantage to having high openness; this reflects the higher payoffs that can be obtained through cooperation with distant others. Thus, the optimal strategy is high openness when infection probability is low. The bottom right‐hand panel shows that when the probability of infection is high for widely separated agents, relatively higher payoffs are received by agents with the lowest openness values—the optimal strategy has changed as the environment has changed, and payoffs are lower overall. Thus, the optimal strategy is low openness when infection probability is high. This first simulation, while simple, illustrates the trade‐off at the heart of the OPTO model: The benefits of high openness disappear and then eventually reverse as the probability of infection in the environment increases.

*Simulation 2: Learning to be open in different environments*. Next, we allow individual agents to adjust their values of openness throughout the simulation. Each time an agent interacts with another agent it adjusts its openness to an extent that depends on the size of the payoff received. If the agent receives a positive payoff at time *t*, it adjusts its openness parameter upward to an extent that is proportional to the size of the positive payoff, as follows:

(4) $$O_{i}^{t+1}=O_{i}^{t}+\alpha *\frac{\left(1-O_{i}^{t}\right)*{\text{Pay}}_{i}^{t}}{A}$$

where *α* is a learning rate (here set to 0.2), and ${\text{Pay}}_{i}^{t}$ is the payoff received by agent *i* at time *t*. The denominator of *A* (here *A *=* *10) normalizes the adjustment relative to the maximum possible positive payoff that can be obtained when both agents cooperate and are maximally distant. (From Eq. (1), the maximum positive payoff is 1 + *P*, so *A *=* *1 + *P*.)

If an agent receives a negative payoff, it adjusts the value of its openness parameter afterward, as follows:

(5) $$O_{i}^{t+1}=O_{i}^{t}-\alpha *\left(1+O_{i}^{t}\right)*\frac{\left(1-O_{i}^{t}\right)*{\text{Pay}}_{i}^{t}}{B}$$

where again the adjustment is normalized by *B* to be relative to the maximum possible loss (here *B *=* *53, as this is the maximum possible negative payoff for *i* in the unfortunate event for agent *i* that agent *i* cooperates, *j* defects, and *i* becomes infected hence receiving both the sucker's payoff of −3 and the infection‐related payoff of −50).

**Figure 2:**
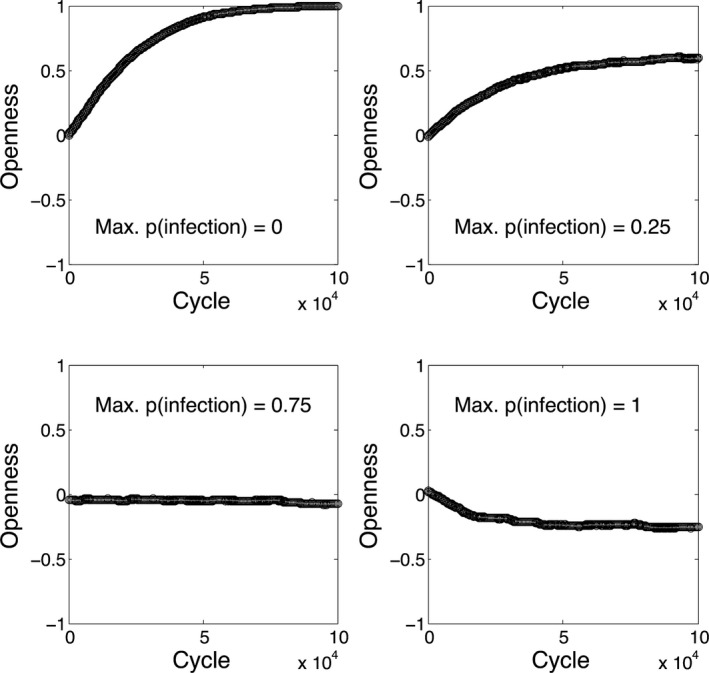
Effects of learning on openness for different values of the probability of infection associated with most distant neighbors.

Fig. 2 shows how the average level of openness within the network changes over cycles of learning in environments with different probabilities of infection. When infection is absent, agents gradually learn to become more open (top left panel), while when the probability of infection is high, agents gradually become less open and prefer to cooperate only with nearby others. While again straightforward, and with effect sizes that are inevitably parameter dependent, the simulation confirms that a very simple learning mechanism can cause the “personalities” of agents to change in response to differing levels of parasitic stress within their environments. Such learning in real populations could occur over various timescales and could involve evolution and/or cultural transmission; we remain neutral on this important point.

*Simulation 3: Emergence of group structure*. Finally, and of particular interest for present purposes, we examined the emergence of group structure in the network. Gray et al. (2014) demonstrated that mutually cooperative groups could evolve in social networks as a result of simple mechanisms involving reciprocity and transitivity, even in the absence of any pre‐existing differences between the agents. Our concern here is with the nature of the groups that form, and in whether they differ as a function of prevailing levels of parasitic infection. For example, when risk of infection is high and increases with the geographical distance separating a pair of interacting agents, intuition suggests that any mutually cooperative groups that form will tend to be composed of agents that occupy spatial locations close to one another. When infection risk is low, in contrast, mutually cooperative groups that include spatially separated agents may be more likely to form. Such behavior would shed light on one mechanism that, according to the parasite stress hypothesis, leads to a more inward‐looking and xenophobic social organization when parasite stress is high. The aim of the present simulation is to determine whether such behavior can occur.

We incorporated the reciprocity and transitivity mechanisms used by Gray et al. (2014). Reciprocity works as follows. If both agents cooperate, their affinity increases, while if both players defect, their affinity reduces (reciprocity). If one defects and one cooperates, their affinity remains unchanged.^8^ The rate of learning is given by an adjustment rate parameter β, here set to 0.5.

Specifically, when mutual cooperation occurs, affinity is increased as follows:

(6) $$C_{t+1}=C_{t}+\beta *\left(1-C_{t}\right)$$

When mutual defection occurs, affinity is reduced as follows:

(7) $$C_{t+1}=C_{t}*\left(1-\beta \right)$$

Transitivity is the tendency for affinity values to adjust toward those of another agent. If both players cooperate, they compare their affinities with all other agents in the network, and the agent with the “weaker” opinion regarding another agent (i.e., with an affinity value nearest to the starting value of 0.5) adjusts its affinity to the other agent by an adjustment factor, as follows (see Gray et al., 2014, for details). If the affinity between agent *i* and agent *k* (*k* being a “friend” of agent *j*) is > 0.5, then:

(8) $$C_{i,k}^{t+1}=C_{i,k}^{t}+\delta *\left(1-C_{i,k}^{t}\right)$$

where δ is an adjustment rate parameter (here set to 0.25). If the affinity between agent *i* and agent *k* is < 0.5, then:

(9) $$C_{i,k}^{t+1}=C_{i,k}^{t}-\delta *C_{i,k}^{t}$$

**Figure 3:**
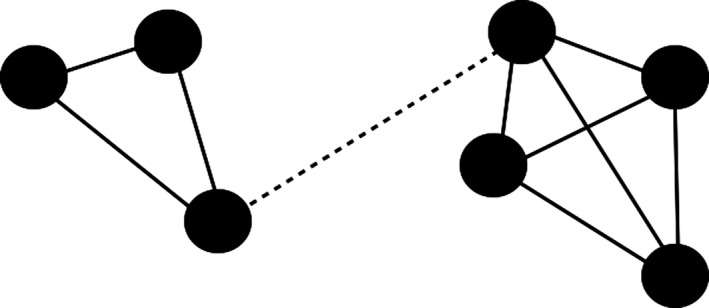
Illustration of a simple network with two sub‐groups.

The main interest here is in the nature of the groups (if any) that form as a result of iterative application of Eqs (6) through (9). Gray et al. (2014) measured group formation using the clustering coefficients of the network; here we adopt a different method. The essence of identifying groups is to determine what division of agents into groups effectively maximizes the ratio of within‐group to between‐group connections. For example, consider the toy network shown in Fig. 3. Each line between two agents shows that those agents are connected (in the present model we say that two agents are “connected” if their mutual affinity is > 0.5, and “not connected” otherwise^9^). The illustrated network divides naturally into two sub‐groups—there is only one connection (the dotted line) between the two obvious sub‐groups, whereas each agent within the sub‐groups is connected to at least two other agents within the same group. Group detection algorithms typically work by successively deleting the connections that have the highest “betweenness” (i.e., participation in the highest number of shortest node‐to‐node pathways), as these tend to be the connections that join what would otherwise be separate groups (Girvan & Newman, 2002). Such a process would eventually lead to each node being a separate “group,” so the process terminates upon finding the partition of network nodes into communities that give the highest value of the statistic normally referred to as the modularity, *Q*, which effectively measures the number of within‐group relative to between‐group connections (Newman & Girvan, 2004). While the measure is computationally expensive to calculate,^10^ it offers an independent index of the number of separate groups into which a network divides.

As the group detection algorithm is computationally intensive, we used a smaller (8 × 8) network for these simulations. In each simulation, we gave all agents the same value of openness and examined what type of group structure emerged. The value of openness was varied across simulations. We examined both the number of separate sub‐groups that were obtained (i.e., the number of sub‐groups that maximized *Q*), although we ignored cases where the highest level of *Q* was associated with the whole network being just a single group. We examined not just the number of groups that emerged but also their structure; specifically, we examined the average spatial distance that separated agents within each group.

**Figure 4:**
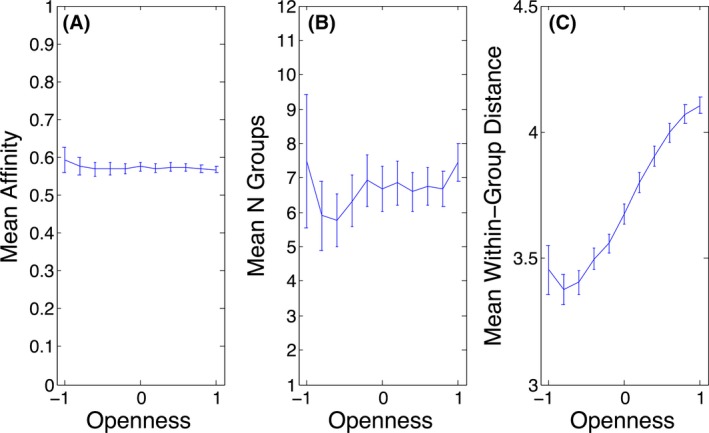
Effects of openness on (A) mean affinity, (B) mean number of groups (excluding single groups), and (C) mean within‐group distance (excluding groups containing only one member).

Results are shown in Fig. 4. The results plotted represent the averages, with associated standard error bars, obtained from 50 replications of a simulation with the relevant parameters; each simulation was run for 2,000,000 time cycles. Panel A shows the average affinity of all agents at the end of simulations which varied in the openness of agents (openness was held constant within each simulation). The panel shows that the average affinity to which agents converge, at just over 0.5, was unaffected by the openness of the agents in the network.

However, high openness was associated with the emergence of a roughly equal number of cooperative groups by the end of simulations (panel B). Thus, typically 6 or 7 separate groups had formed by the end of simulations (recall there were 64 agents in all). The relationship shown in panel B is, however, highly dependent on parameters such as learning rate, the number of simulation cycles completed, and outliers.

Of most interest, we investigated whether the groups that had formed by the end of each simulation run showed greater assortativity (here defined as a shorter average spatial distance between group members) when the agents' openness was lower. Panel C shows the average within‐group distance as a function of the openness of agents. This relationship turns out to be fairly robust to parametric variations. Note that the average within‐group distance for randomly selected groups would be four. Thus, panel C shows that low levels of openness lead to the formation of groups that include geographically more similar neighbors than would be found in a randomly selected group, whereas high values of openness are associated with much more heterogeneous groups, with values > 4.0 effectively indicating negative assortativity.

Thus, the simulation shows that the type of mutually cooperative social group that emerges when openness is low (which will be the case when levels of parasitic stress are high, as shown in Simulation 2) are more inward looking and highly assortative. Thus, the agent‐based model illustrates a mechanism by which reduced openness (Simulation 2) and hence greater assortativity (Simulation 3) may emerge as a consequence of high levels of infection within an environment.

## 3 Discussion

Overall, we interpret the results as supportive of the OPTO model. The model illustrates mechanisms by which the development of cooperation with out‐groups, in turn associated with more liberal ideology and open personality styles, may be facilitated when levels of infection are low. Conversely, if perceived levels of infection‐related threat increase within a local region (e.g., as a result of climate change), a more conservative cognitive, ideological, and personality style may result—leading in turn to reduced levels of cooperation with out‐groups and hence increased conflict and difficulty in effecting global change. Indeed, the frequency of the outbreak of interstate conflicts and civil war has been attributed to the intensity of infectious disease across countries of the world (Letendre, Fincher, & Thornhill, 2010, 2012; although see Hendrix & Gleditsch, 2012). Thus, in regions where local warming may increase parasite prevalence or decrease health through other means, increasingly ethnocentric norms may emerge.

Our model is simplistic in a number of ways. There is of course much more to political ideology than can be captured by dimensions such as openness and xenophobia. The notion of openness that we implement is purely relative in that agents learn to interact preferentially with either distant or close others; there is in the present version of the simulation no mechanism for overall changes in openness such that an agent could become more or less likely to interact with all other agents irrespective of distance. Also, we have focused on the formation of social groups as a result of learning, and remained neutral on the multiple scales (e.g., evolutionary, social, individual) over which such changes may occur; we assume, for example, that initial allelic differences in infection vulnerability and differential ideology manifestation may influence group formation. It is also clear that quite short‐term effects can be seen, as when disgust‐related primes lead to increased conservatism (Helzer & Pizarro, 2011; Terrizzi et al., 2010) or when an outbreak of infectious disease such as avian flu leads to “othering” of out‐group members such as migrant workers in mainland China (Goodwin, Haque, Neto, & Myers, 2009).

Moreover, it is undoubtedly the case that the structure and norms of society influence the individual as well as the reverse. We have focused here on specifying a mechanism by which a key feature of the environment (parasitic stress) may affect a characteristic of the individual (openness), which in turn influences the social group structures that evolve. However, the structure of the society inhabited by an individual may of course influence the characteristics of that individual via social norms and cultural transmission. Moreover, openness may be more adaptive (even when parasitic stress is held constant) when supportive social structures, such as government‐provided healthcare or general security and abundance of resources, may lead to different parental investment strategies and/or reduce the need to depend on in‐groups for help when it is needed (see Cashdan & Steele, 2013; Hackman & Hruschka, 2013; Hruschka & Henrich, 2013). A complete model will need to capture the dynamic interplay of these factors over time.

The possibility that (perceived or actual) levels of parasitic infection may be causally related to changes in cognition and ideology is of particular relevance in light of current evidence for global warming. For example, the ability of mosquitoes to transmit malaria is temperature dependent (Mordecai et al., 2013), with suggestions that malaria risks will increase with global warming (Martens et al., 1999; although see Rogers & Randolph, 2000). There is thus a risk that undesirable positive feedback loops emerge, such that increased perception of environmental threat will engender an ethnocentric perspective that reduces the level of out‐group trust and cooperation that would be needed to mitigate the threat.

## Notes

1 We use the terms “parasite,” “pathogen,” and “infectious disease” interchangeably and follow the literature in using these terms to refer to any disease‐causing pathogens, including, for example, viruses, bacteria, and helminths, and we consider infectious diseases independently of their transmission vector (e.g., insect, air, water, food, direct contact) to be relevant.

2 Tybur et al. (2010) find no relationship between individuals' sensitivity to pathogen disgust and their political conservatism, but results may differ when purely social conservatism is examined. Moreover, priming effects and group‐level associations could coexist with the absence of relationships at an individual level.

3 Of course, there is much more to political liberalism than the “openness to out‐groups” aspect that forms the present focus; the aim here is to examine the possible origins of a core component of social conservatism.

4 Gray et al. (2014) use the term “closeness”; instead, we use the term “affinity” to avoid confusion with the notion of geographical/spatial closeness that we introduce.

5 Although full connectivity is not plausible as a model of human social networks, it suffices to illustrate the points we wish to make here.

6 From this point we will not always use the “exotic” qualifier, but it should be understood.

7 This is done to ensure that all distances have a reasonable chance of being selected, to speed up the simulations, and to avoid the formation of local groups due to propinquity‐based sampling; results are qualitatively similar if randomly selected pairs of agents are selected without regard to their separation.

8 It might be argued on intuitive grounds that affinity would change asymmetrically if one agent cooperates and the other defects. However, we have preserved this feature of the Gray et al. (2014) model to avoid adding considerable additional complexity which would not illuminate the issue at hand.

9 Although this discards information about strength of affinity, affinities tend toward 0 or 1 over time (because of transitivity), and it is natural to think of group co‐membership as a binary construct. In any case, we found it was not computationally feasible to use group‐detection algorithms based on continuous strength measures.

10 We used the script “newman_comm_fast.m”; it is available from http://strategic.mit.edu/downloads.php?page=matlab_networks.
